# Comparative Metagenomic Profiling of Symbiotic Bacterial Communities Associated with *Ixodes persulcatus*, *Ixodes pavlovskyi* and *Dermacentor reticulatus* Ticks

**DOI:** 10.1371/journal.pone.0131413

**Published:** 2015-07-08

**Authors:** Alexander Kurilshikov, Natalya N. Livanova, Nataliya V. Fomenko, Alexey E. Tupikin, Vera A. Rar, Marsel R. Kabilov, Stanislav G. Livanov, Nina V. Tikunova

**Affiliations:** 1 Institute of Chemical Biology and Fundamental Medicine SB RAS, Novosibirsk, Russia; 2 Institute of Systematics and Ecology of Animals SB RAS, Novosibirsk, Russia; University of Kentucky College of Medicine, UNITED STATES

## Abstract

*Ixodes persulcatus*, *Ixodes pavlovskyi*, and *Dermacentor reticulatus* ticks inhabiting Western Siberia are responsible for the transmission of a number of etiological agents that cause human and animal tick-borne diseases. Because these ticks are abundant in the suburbs of large cities, agricultural areas, and popular tourist sites and frequently attack people and livestock, data regarding the microbiomes of these organisms are required. Using metagenomic 16S profiling, we evaluate bacterial communities associated with *I*. *persulcatus*, *I*. *pavlovskyi*, and *D*. *reticulatus* ticks collected from the Novosibirsk region of Russia. A total of 1214 ticks were used for this study. DNA extracted from the ticks was pooled according to tick species and sex. Sequencing of the V3-V5 domains of 16S rRNA genes was performed using the Illumina Miseq platform. The following bacterial genera were prevalent in the examined communities: *Acinetobacter (all three tick species)*, *Rickettsia (I*. *persulcatus* and *D*. *reticulatus)* and *Francisella (D*. *reticulatus)*. *B*. *burgdorferi* sensu lato and *B*. *miyamotoi* sequences were detected in *I*. *persulcatus* and *I*. *pavlovskyi* but not in *D*. *reticulatus* ticks. The pooled samples of all tick species studied contained bacteria from the Anaplasmataceae family, although their occurrence was low. DNA from *A*. *phagocytophilum* and *Candidatus* Neoehrlichia mikurensis was first observed in *I*. *pavlovskyi* ticks. Significant inter-species differences in the number of bacterial taxa as well as intra-species diversity related to tick sex were observed. The bacterial communities associated with the *I*. *pavlovskyi* ticks displayed a higher biodiversity compared with those of the *I*. *persulcatus* and *D*. *reticulatus* ticks. Bacterial community structure was also diverse across the studied tick species, as shown by permutational analysis of variance using the Bray-Curtis dissimilarity metric (*p* = 0.002). Between-sex variation was confirmed by PERMANOVA testing in *I*. *persulcatus* (*p* = 0.042) and *I*. *pavlovskyi* (*p* = 0.042) ticks. Our study indicated that 16S metagenomic profiling could be used for rapid assessment of the occurrence of medically important bacteria in tick populations inhabiting different natural biotopes and therefore the epidemic danger of studied foci.

## Introduction

Ticks belonging to the genera *Ixodes* and *Dermacentor* (the family Ixodidae) are capable of transmitting a number of bacteria and viruses to humans, including causative agents of Lyme disease, rickettsioses, ehrlichioses, relapsing fever borreliosis, tularemia, Q fever, and tick-borne encephalitis [1,2,3]. In Western Siberia, *I*. *persulcatus* ticks are the main vector for tick-borne encephalitis and Kemerovo viruses [4,5,6] and many bacterial agents including *Borrelia burgdorferi* sensu lato, *B*. *miyamotoi*, “*Candidatus* Rickettsia tarasevichiae”, *Anaplasma phagocytophilum*, *Ehrlichia muris*, and *Bartonella* spp. [7,8,9,10,11,12,13,14]. *I*. *pavlovskyi* ticks inhabit the southern part of Western Siberia (Altai, Kuznetsk Alatau, and Salair mountains and foothill areas) and are responsible for transmission of infectious agents of Lyme disease and a tick-borne encephalitis virus [15,16]. Since the beginning of this century, *I*. *pavlovskyi* ticks have been abundant in the green belts of the Novosibirsk and Tomsk cities, which are located more northward in the Western Siberian Plain, and currently, this tick species is more prevalent compared with the *I*. *persulcatus* ticks and accounts for 82 and 90% of the collected samples, respectively [17,18,19]. A third Ixodidae tick species, *Dermacentor reticulatus*, is abundant in Western Siberia and is involved in the transmission of etiological agents of human and animal tick-borne diseases, such as Omsk hemorrhagic fever virus, *B*. *burgdorferi* s. l., *Rickettsia raoultii*, *Bartonella quintana*, and *B*. *canis* [11,14,20,21].

Recently, new microorganisms associated with human infections have been detected in different Ixodidae tick populations in the Asian region of Russia [6,9,12,22]; however, it is likely that a number of infectious agents are still unknown. Currently, 16S profiling is among the most powerful tools for examining microbial communities. This method uses polymerase chain reaction (PCR) with primers specific to the conserved regions of 16S rRNA genes to obtain a set of the corresponding DNA fragments. High-throughput sequencing of the amplicons is performed using second-generation sequencing platforms [23]. Analysis of the 16S rRNA gene sequences allows for phylotyping of the prokaryotes, including the organisms unable to be cultivated [24], which is especially important when analyzing symbiotic communities. This approach has been successfully used to examine the *I*. *ricinus* microbiome and allowed for the detection of various infectious agents within this species, including *Borrelia* species, *Rickettsia* spp., *A*. *phagocytophilum*, *Ehrlichia* spp., and “*Candidatus* Neoehrlichia mikurensis” [25,26,27]. Methods including 16S profiling were also applied for examination of microbial diversity in *Amblyomma americanum*, the most commonly encountered tick species in the southeastern-central USA [28,29], and it was found that some members of the bacterial community within *A*. *americanum* change during the tick life cycle. Furthermore, microbiomes from the salivary glands of *Ixodes ovatus*, *I*. *persulcatus* and *Haemaphysalis flava* ticks inhabiting Japan were examined [30]. This study indicated that bacterial communities in tick microbiomes were different between tick species [30].

*I*. *persulcatus*, *I*. *pavlovskyi*, and *D*. *reticulatus* are abundant in the green belts of large Siberian cities, agricultural areas, and popular tourist sites, and little is currently known about the bacterial communities associated with these ticks which are able to transmit a number of infectious agents and frequently attack people and home animals, so the goal of this study was to assess the taxonomic structure of *I*. *persulcatus*, *I*. *pavlovskyi*, and *D*. *reticulatus* microbiomes using high-throughput sequencing.

## Methods

### Tick collection and sample preparation

Adult ticks were collected by flagging from three sampling sites in the Novosibirsk region of Russia (Table 1). *I*. *persulcatus* ticks were flagged in site 1 (forest-steppe, Toguchin district); *I*. *pavlovskyi* and *D*. *reticulatus* ticks were flagged in sites 2 and 3, respectively (forest suburbs of Novosibirsk, Novosibirsk district). Coordinates of the sampling sites are presented in Table 1. Each tick species dominated in the selected site. No specific permission was required for our activities in locations involved in the study. Our field activities did not involve endangered or protected species. Ticks were sampled from 24 April to 30 May 2012 during the peak activity period for these Ixodidae ticks. Live ticks were taxonomically characterized by microscopic analysis using standard taxonomic keys. The relative abundance was assessed as the number of ticks collected per 1 km. Before study, ticks were stored at -70°C. A total of 1214 ticks were used for this study (*I*. *persulcatus*, n = 414; *I*. *pavlovskyi*, n = 436; *D*. *reticulatus*, n = 364).

**Table 1:** Library description.

Species	*Ixodes persulcatus*				*Ixodes pavlovskyi*				*Dermacentor reticulatus*			
Flagging site	Site 1				Site 2				Site 3			
Site description	forest-steppe				forest suburbs				forest suburbs			
Site coordinates	N55°01'07'', E84°06'41''				N54°49'21'', E83°06'41''				N54°53'15'', E83°08'45"			
Sex	male		female		male		female		male		female	
Library name	PersM.1	PersM.2	PersF.3	PersF.4	PavlM.5	PavlM.6	PavlF.7	Pavl.F8	DermM.9	DermM.10	DermF.11	DermF.12
Number of ticks in library	120	120	87	87	120	120	98	98	89	88	94	93
Number of reads in library	5168	5806	14043	11884	12796	12999	19161	12466	5322	9372	12639	10992

### DNA extraction and sequencing

Before DNA extraction, ticks were washed in distilled water, 70% ethanol and distilled water as described previously [25]. Then ticks were grouped according to their species and sex; each group consisted of eight *I*. *persulcatus* or *I*. *pavlovskyi* males, or up to five *I*. *persulcatus*, *I*. *pavlovskyi* females, or up to five *D*. *reticulatus* ticks. Ticks within each group were frozen (30 min/ – 80°C), macerated with a sterile micropestle, and total DNA was extracted using the Proba NK kit (DNA-technology, Russia) according to the manufacturer’s instruction. This kit provides DNA extraction from 100 mkg specimen. Aliquots of DNA from each group were subsequently combined resulted in 12 pooled samples (persM.1, persM.2, persF.3, persF.4, pavlM.5, pavlM.6, pavlF.7, pavlF.8, dermM.9, dermM.10, dermF.11, and dermM.12) providing two biological replicates for each ‘one species-one sex’ combination. Each pooled sample included 88–120 males or 93–98 females (Table 1).

The presence of bacterial DNA was assessed by PCR using primers that specifically amplified the hypervariable regions V3-V5 of 16S rRNA genes [24]. 10 ng of DNA was used as a template in PCR. To prevent contamination, we performed DNA isolation, PCR master mix assembly, and amplification in separate rooms. Aerosol-free pipette tips were used at each stage. We included negative control reactions with bi-distilled water in each experiment.

Fusion primers comprised of the fragment-specific 3’ end, barcode, and adapter sequence for the Illumina MiSeq platform (Illumina) were used for amplification. The length of the amplified fragment was approximately 430 bp. Sequencing by paired-end reads with a read length of 250 nucleotides was conducted on the Illumina MiSeq platform at the Genomics Core Facility of the Siberian Branch of the Russian Academy of Sciences.

### Data analysis

Paired-end reads from Illumina Miseq platform were assembled using Pandaseq v2.4. Reads containing more than six ambiguous bases were eliminated from the dataset [31]. The pooled samples were taxonomically characterized using the QIIME v. 1.7.0 software package [32]. Analysis included read clustering by the UCLUST software package with a minimum similarity coefficient of 97% [33]. Representative sequences for each operational taxonomic unit (OTU) were selected. Taxonomic classification for each representative was performed using the RDP classifier with 80% confidence [34]. Taxonomic trees were constructed using the MEGAN software package.

Hierarchical clustering, between-class analysis and diversity analysis were performed using the R programming language. Hierarchical clustering of pooled samples was performed using hclust and visualized as a heatmap. Diversity analysis was completed using the “vegan” package [35]. Between-class analysis was performed using the package “ade4” with euclidean and UniFRAC distances [36]. The Permutational Multivariate Analysis of Variance (PERMANOVA) function from package “vegan” was used to assess the effect of gender and species on taxonomic profiles. The permuted p-value was obtained by the Bray-Curtis dissimilarity measure for 10 000 permutations.

## Results

### Bacterial diversity estimation

The 16S rRNA gene fragments used for phylotyping contained V3, V4 and V5 hypervariable regions. These regions allow for effective genus-level classification of bacteria [24,37]. A total of 12 pooled samples were sequenced, resulting in twelve libraries. The number of reads per library was 6152 to 23 477 (Table 1). To avoid biodiversity inflation caused by sequencing errors and PCR chimeras, all OTUs with single observation count were removed from data. In total, 25767 OTUs were singletons while 2802 OTUs were presented in two or more copies. DNA sequences from 726 OTUs were detected in the *I*. *persulcatus* ticks (520 in male ticks and 390 in female ticks); 2306 OTUs in the *I*. *pavlovskyi* ticks (1709 in males and 1316 in females); and 654 OTUs in the *D*. *reticulatus* ticks (474 in males and 437 in females). Extrapolated richness of the bacterial communities from the examined groups was assessed using chao and bootstrap estimators (Table 2). Because of the different data volumes for each library, the samples were subsampled according to the read number in the smallest library. Shannon index and beta diversity index were calculated on rarefied data, as well. This estimation proved that the bacterial communities detected in *I*. *pavlovskyi* ticks were the most diverse, containing 2058 ± 232 OTUs and Shannon index of 3.86 ± 0.73. The *D*. *reticulatus* and *I*. *persulcatus* microbiomes were less diverse, comprised of 637 ± 65 OTUs (*H* = 2.01 ± 0.23) for *D*. *reticulatus*, and 773 ± 98 OTUs (*H* = 2.06 ± 0.27) for *I*. *persulcatus* ticks.

**Table 2:** Diversity analysis.

Library number	PersM.1	PersM.2	PersF.3	PersF.4	PavlM.5	PavlM.6	PavlF.7	PavlF.8	DermM.9	DermM.10	DermF.11	DermF.12
Tick species	*Ixodes persulcatus*				*Ixodes pavlovskyi*				*Dermacentor reticulatus*			
Tick sex	male		female		male		female		male		female	
**Per-group diversity statistics**												
Number of OTUs per group	520		390		1709		1316		474		437	
Number of OTUs after rarefaction	511		252		1233		792		400		307	
Extrapolated richness (Chao)	699 ± 28		367 ± 24		1721 ± 47		1182 ± 46		558 ± 27		404 ± 19	
Extrapolated richness (bootstrap)	598 ± 88		297 ± 47		1447 ± 214		937 ± 146		470 ± 71		357 ± 51	
Shannon index (*H*)	2.29 ± 0.06		1.83 ± 0.03		4.48 ± 0.05		3.23 ± 0.24		2.18 ± 0.18		1.83 ± 0.05	
Beta diversity	0.52		0.56		0.53		0.57		0.53		0.49	
**Per-species diversity statistics**												
Tick species	*Ixodes persulcatus*				*Ixodes pavlovskyi*				*Dermacentor reticulatus*			
Number of OTUs per species	726				2306				654			
Number of OTUs after rarefaction	638				1693				532			
Extrapolated richness (Chao)	973 ± 46				2749 ± 89				849 ± 50			
Extrapolated richness (bootstrap)	773 ± 98				2058 ± 232				637 ± 65			
Shannon index (*H*)	2.06 ± 0.27				3.86 ± 0.73				2.01 ± 0.23			
Beta diversity	1.56				1.59				1.28			

### Sample clustering and community structure

**Fig 1:**
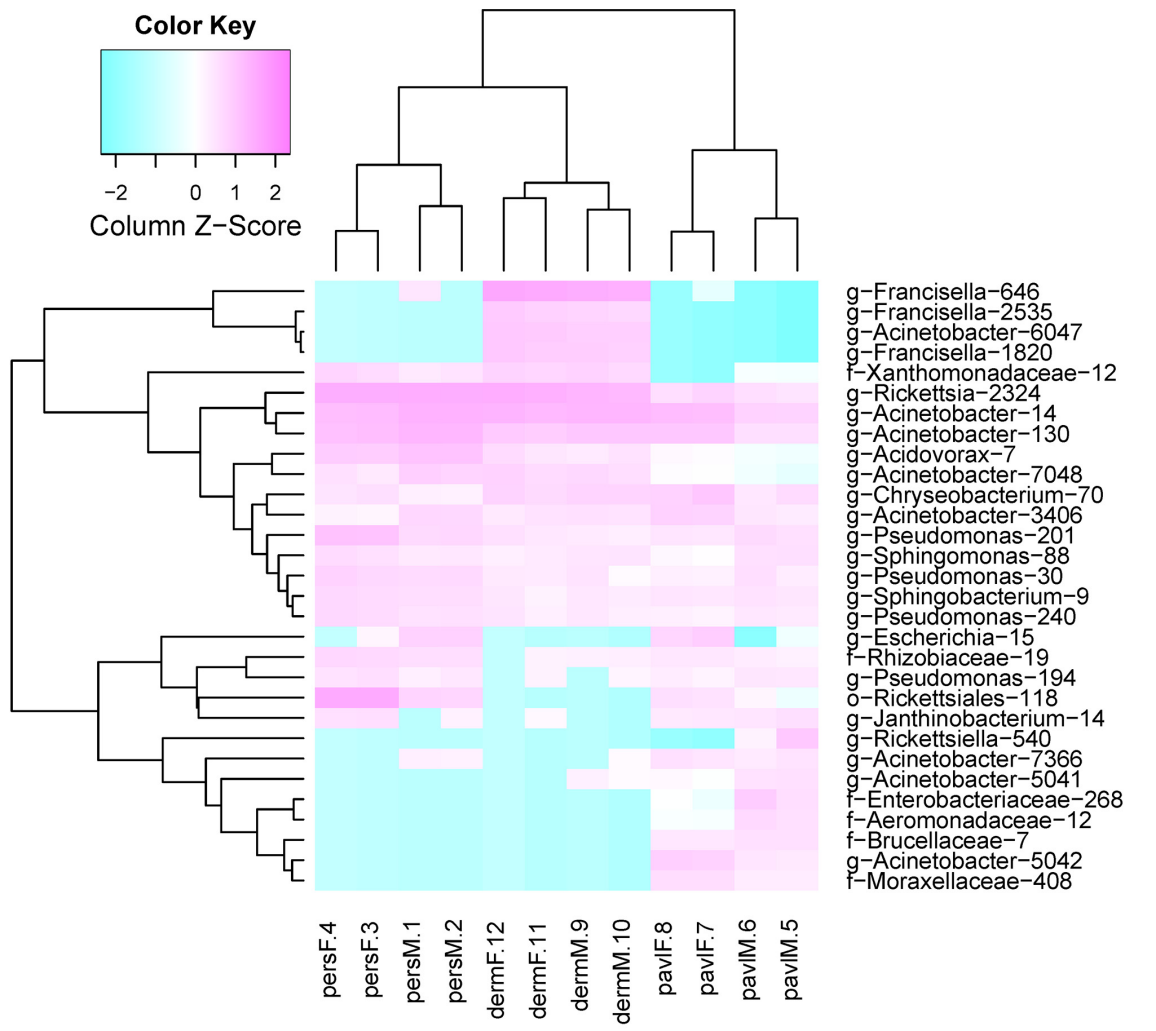
Heatmap based on 30 mostly reprepreted OTUs. Heatmap was constructed using euclidean distance method for log-scaled in-sample taxon share values. Sample names on the X axis consist of tick species, sex and library number. OTU names on the Y axis contain OTU's lowest common ancestor name and unique integer OTU key.

**Fig 2:**
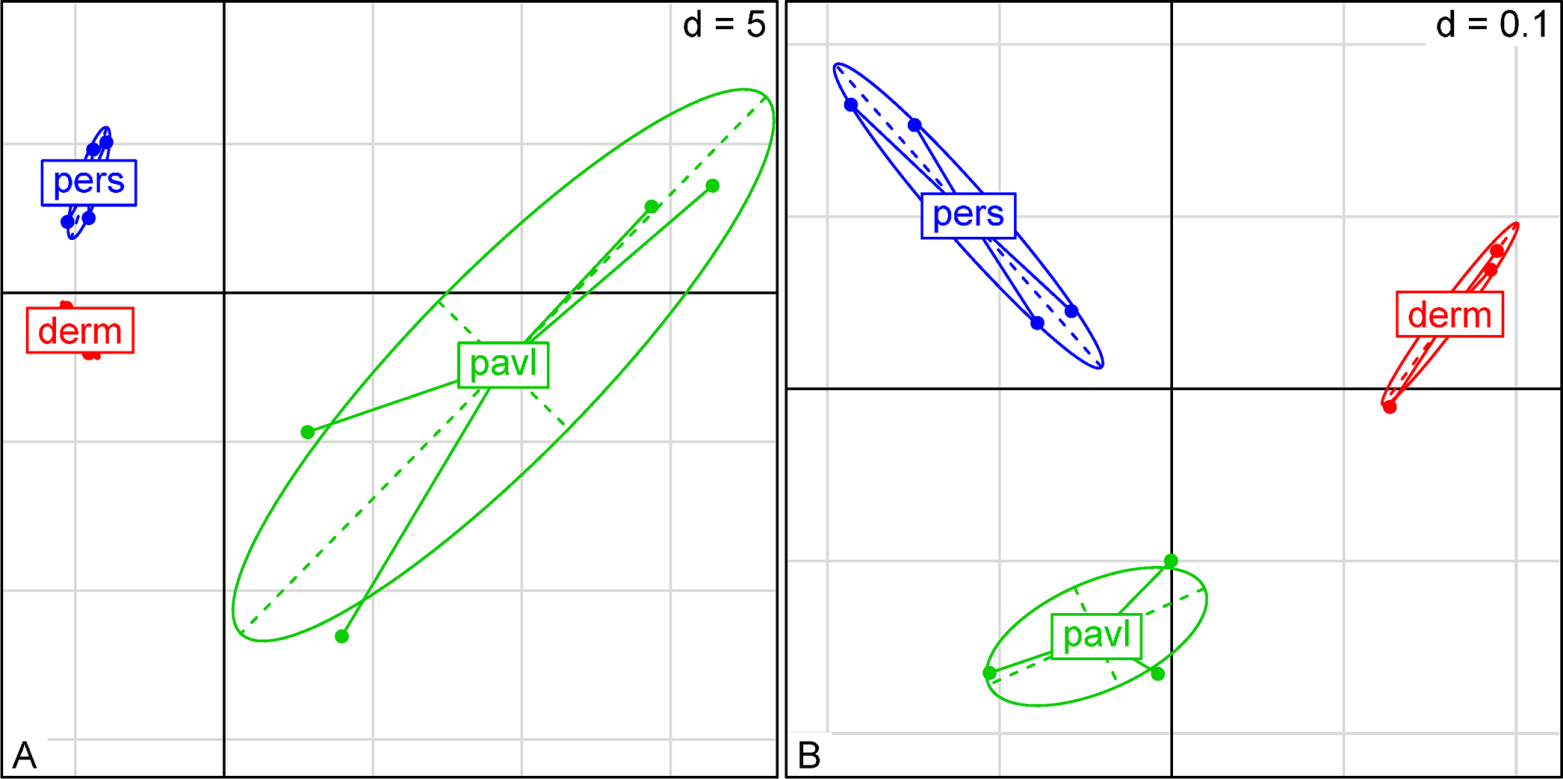
Between-class analysis plots for taxon-based distances. A) MDS plot based on PCA for 150 taxa with the largest share. Principal component analysis was performed using package “ade4” with data scaling and centering. B) MDS plot based on the UniFRAC distance. Calculation of UniFRAC distance was performed using QIIME package. First and second principal components describe 40.69% and 32.82% of total variance respectively.

To detect differences in microbiome structures among different tick species, hierarchical clustering and between-class analysis were used. The most frequently represented OTUs in the pooled samples were used in hierarchical cluster analysis based on a distance matrix (Fig 1). Between-class analysis based on the taxonomic composition demonstrated that the species-level differences in the examined samples are detectable within the first two variation components (Fig 2). The intra-species variation within samples from the *I*. *persulcatus* and *D*. *reticulatus* ticks were relatively small versus that of *I*. *pavlovskyi*. A strict separation of the studied tick groups according to the tick species criterion was observed. When using the PCoA analysis with weighted unifrac distance measures, the first two components account for 73.51% of the total variation and provide for a more strict separation of the groups (Fig 2). PERMANOVA analysis based on in-sample OTU amounts also indicates a strict separation in microbiome structure among these three tick species (F-stat = 5.323, df = 2, *p* = 0.02).

Sex-specific differences in the studied microbiomes were detected. Using distance matrix with euclidean distances, it was shown that microbiome of *D*. *reticulatus* ticks was the most homogeneous across all pooled samples while the sex-specific differences within the *I*. *pavlovskyi* and *I*. *persulcatus* samples were more significant (Fig 1). Within-group variations across the studied samples were varied. The male *I*. *persulcatus* and female *D*. *reticulatus* groups displayed the least intragroup variation versus the male and female *I*. *pavlovskyi* ticks that had a considerably higher variation value. PERMANOVA analysis based on in-sample OTU amounts shows that sex-specific variations in microbiome structure were substantial in the *I*. *persulcatus* (F-stat = 11.841, df = 1, *p* = 0.042) and *I*. *pavlovskyi* samples (F-stat = 4.0852, df = 1, *p* = 0.042) and insignificant in the *D*. *reticulatus* samples (F-stat = 2.7301, df = 1, *p* = 0.333).

### Taxonomic composition

Occurrence of the detected taxa is shown on the MEGAN taxonomic tree (S1 Table, S1 Fig). The bacterial DNA sequences of Alpha- and Gammaproteobacteria types were prevalent in all of the assayed pooled samples with 30.2% and 60.8% average occurrence, respectively. The most abundant families were Rickettsiaceae (20.5%, on average, varying from 1.5% in *I*. *pavlovskyi* to 21.1% in *D*. *reticulatus* and 41.1% in *I*. *persulcatus*), Francisellaceae (16.7–55% in *D*. *reticulatus)* and Moraxellaceae (from 9% to 76% in *I*. *persulcatus*).

**Fig 3:**
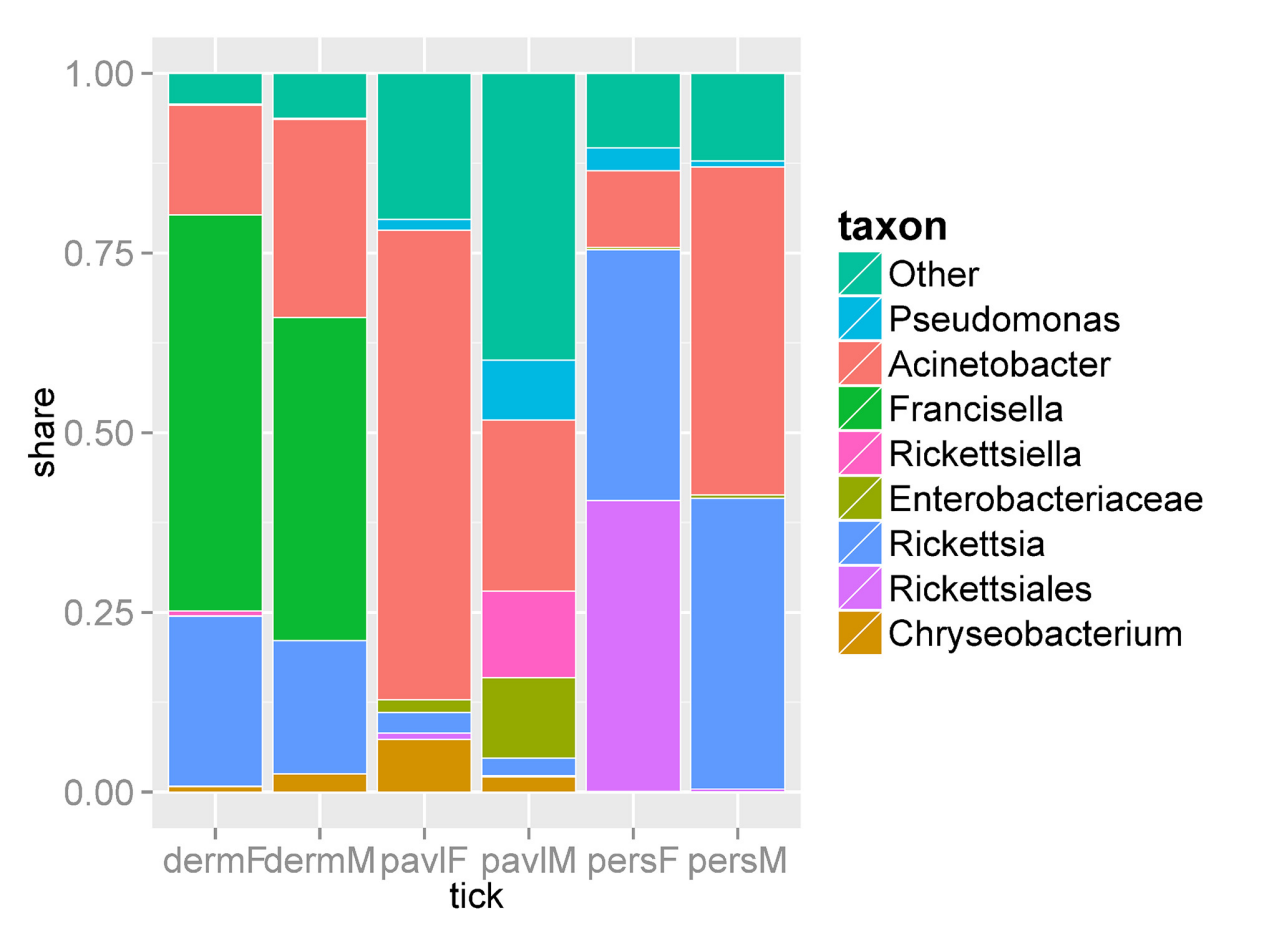
Bar graph with the share of the 8 most represented taxa. Share of Rickettsiales order and Enterobacteriaceae family cover only hits that hadn't be included in the lower levels of these taxa.

DNA sequences belonging to the bacterial genera *Acinetobacter* (Gammaproteobacteria, Pseudomonadales, Moraxellaceae) and *Rickettsia* (Alphaproteobacteria, Rickettsiales, Rickettsiaceae) were most abundant in all samples; however, the abundances of these groups in the *I*. *persulcatus*, *I*. *pavlovskyi*, and *D*. *reticulatus* bacterial communities varied considerably. *Acinetobacter* bacteria were prevalent in the female *I*. *pavlovskyi* (65.4%) and male *I*. *persulcatus* (45.6%) ticks and ranged from 8.8 to 31.3% in the microbiomes of the remaining tick groups (Fig 3, S1 Table, S1 Fig). In addition to *Acinetobacter* spp., other members of the order Pseudomonadales and families Moraxellaceae (*Enhydrobacter* spp., *Psychrobacter* spp., etc.) and Pseudomonadaceae (*Pseudomonas* spp.) were also detected. The occurrence of bacteria belonging to the genus *Rickettsia* was higher in the *I*. *persulcatus* (males, ~41%, and females, ~35%) and *D*. *reticulatus* (males, 18.5%, and females, 23.7%) ticks (Fig 3, S1 Fig), but considerably lower in *I*. *pavlovskyi* ticks, amounting to 2.5 and 3.8% in males and females, respectively. In addition, DNA from the Rickettsiales bacterium “Montezuma” was highly abundant (~ 40%) in the microbiome of female *I*. *persulcatus* ticks, whereas its rate did not exceed 1% in male *I*. *persulcatus* ticks and other pooled samples (Fig 3, S1 Fig).

Bacteria from the *Francisella* genus (Gammaproteobacteria, Thiotrichales, Francisellaceae) were prevalent in the microbiomes of *D*. *reticulatus* ticks, representing approximately 50% of sequences (Fig 3, S1 Table), and were almost absent in the microbiomes of the *Ixodes* ticks. In addition to *Francisella* spp., *Francisella*-like endosymbionts [38] belonging to the family *Francisellaceae* were also detected in *D*. *reticulatus* ticks. The 16S rRNA gene fragments used in this study did not allow discrimination of pathogenic and non-pathogenic *Francisella* species.

DNA sequences of medically significant bacteria belonging to the genus *Borrelia* (Spirochaetes, Spirochaetales, Spirohaetaceae) were present in *I*. *persulcatus* and *I*. *pavlovskyi* ticks (S1 Table, S1 Fig). The selected 16S rRNA locus made it possible to assign borrelias to individual genetic groups. Both *B*. *burgdorferi* s. l. and *B*. *miyamotoi* were detected in the *I*. *persulcatus* and *I*. *pavlovskyi* ticks. No *Borrelia* spp. were found in the *D*. *reticulatus* ticks. Several pooled samples contained medically relevant bacteria from the family Anaplasmataceae (Alphaproteobacteria, Rickettsiales), although their occurrence was low (S1 Table, S1 Fig). DNA sequences from *Ehrlichia* spp. that were most similar to those of *E*. *muris* were detected in *I*. *persulcatus* and *D*. *reticulatus* ticks. *I*. *persulcatus* and *I*. *pavlovskyi* ticks contained *A*. *phagocytophilum* DNA; in addition, DNA sequences from *Candidatus* Neoehrlichia mikurensis were found in *I*. *pavlovskyi* male ticks.

Almost all pooled samples contained small amounts of DNA sequences from *Pseudomonas* spp., *Chryseobacterium* spp., *Acidovorax* spp., *Janthinobacterium* spp., *Sphingobacterium* spp., and other free-living saprophytic bacteria (Fig 3, S1 Table, S1 Fig). Presumably, these bacteria inhabit the tick chitin exoskeleton. Some animal and human symbiotic bacteria—*Bacteroides*, *Prevotella*, *Escherichia*, *Enterobacter*, *Bifidobacterium*, *Yersinia*, and others—were also detected in small amounts (S1 Table, S1 Fig).

In addition, DNA sequences from the bacteria belonging to the genus *Ricketsiella* recently classified as pathogens of arthropods were found in the *D*. *reticulatus* and *I*. *pavlovskyi* ticks [39]. These sequences were present in all *D*. *reticulatus* samples (0.01–0.9%) and males *I*. *pavlovskyi* samples (0.6–23.6%).

## Discussion

The microbiomes associated with ticks contain symbiotic and parasitic species as well as infection agents transmitted by ticks to their vertebrate hosts, whose pathogenicity for ticks is unknown [40,41]. Here, we describe the biodiversity of the bacterial communities associated with the *I*. *persulcatus*, *I*. *pavlovskyi*, and *D*. *reticulatus* ticks that inhabit Western Siberia and frequently attack people and livestock in this region. Ticks were collected from three sampling sites, which are located within the Novosibirsk region and display similar climates typical for Northern forest-steppe area in Siberia. In these sampling sites, larval and nymphal stages of the studied ixodidae ticks are mainly fed on the northern birch mouse (*Sicista betulina*), the striped field mouse (*Apodemus agrarius*), the grey red-backed vole (*Myodes rufocanus*), the northern red-backed vole (*M*. *rutilus*), and the common shrew (*Sorex araneus*). These sampling sites were selected because each tick species was prevalent in the corresponding site and other ixodidae ticks were collected in solitary cases. The relative abundance of *I*. *persulcatus*, *I*. *pavlovskyi*, and *D*. *reticulatus* ticks in sampling sites 1, 2, and 3 in 2012 was 32.4, 31.8, and 41,9 individuals per km, respectively. Other ixodidae tick species were found in corresponding sampling sites in single cases.

More than 1200 ticks of three species were included in this study. To assess the bacterial communities associated with these tick species, 16S metagenomics profiling was performed on twelve pools–four pools for each species (two males and two females). Each pool contained 87–120 ticks. Such tick quantity increases result reliability for large-scale population studies. It should be noted that the prevalence of infectious agents in individual ticks inhabiting Novosibirsk region was studied earlier and it was found that the occurrence of certain infectious agents is low [9,12,14].

We detected both species- and sex-specific differences among the microbiomes of these three tick species. Different clustering algorithms demonstrated that the *I*. *persulcatus*, *I*. *pavlovskyi*, and *D*. *reticulatus* microbiomes differ in both their alpha-diversity levels and taxonomic structures. Taxonomic diversity of the symbiotic microbiomes in the studied ticks was relatively low when compared with that of higher animals and ecological systems. Notably, the symbiotic diversity of the *I*. *pavlovskyi* microbiomes exceeded that of the *I*. *persulcatus* and *D*. *reticulatus* ticks in a statistically significant manner. In addition to alpha-diversity, the examined microbiomes differed in their dominant species and abundances of various subdominants.

Each tick species was collected from a different sampling site associated with different microhabitats that could contribute to the variation in the studied microbiomes. However, these sampling sites are located in one geographic area with similar climate and reservoir host composition, so, we assume that at least a part of the differences among microbiomes of these tick species is species-specific. Earlier analyses of the microbial populations contained in the salivary glands of *I*. *ovatus*, *I*. *persulcatus* and *H*. *flava* ticks collected from the Shizuoka Prefecture of Japan indicated differential bacterial composition among these tick species [30]. However, whether the differences observed in that study and in our work are associated with both tick species and habitats requires further examination. In addition, this study revealed sex-specific differences in the bacterial communities present in the *I*. *persulcatus and I*. *pavlovskyi* ticks. Our data are consistent with results observed in analyses of the microbiome of *A*. *americanum* ticks [29].

Three prevalent bacterial genera—*Acinetobacter*, *Rickettsia*, and *Francisella*—accounted for 68% of the microbiomes of the studied tick species. The *Acinetobacter* bacteria are free-living aerobic saprophytes with oxidative metabolism. Some *Acinetobacter* species are typical representatives of nosocomial infections. These bacteria have been found in arthropods, including ticks [42]. 16S profiling has allowed for the detection of *Acinetobacter* in *I*. *ricinus*, *I*. *ovatus*, *I*. *persulcatus and H*. *flava* ticks in small amounts [27,30], whereas we detected these bacteria in all of the assayed pooled samples in considerably larger amounts from 8.8 to 74% of the microbiome. Other free-living aerobic saprophytes, including various members of the family Pseudomonadaceae, were also detected in all pooled samples examined, though in smaller amounts.

The share of bacteria belonging to the genus *Rickettsia* in the *I*. *persulcatus* and *D*. *reticulatus* microbiomes was large versus that of the *I*. *pavlovskyi* microbiome, in which *Rickettsia* spp. were fewer. It is unclear whether this is determined by a considerable variation in the rate of rickettsia-infected ticks in particular sampling sites or by different tropism of *Rickettsia* spp. to these tick species, including two closely related ixodes ticks species. Earlier investigations demonstrated that the occurrence of *Rickettsia* spp. sequences can be high in both *I*. *persulcatus* and *D*. *reticulatus* [11,31,43]. The 16S rRNA gene fragments used for amplification are highly conserved within the *Rickettsia*, which hinders their species-level identification. Nevertheless, this allowed for detection of DNA indicating the presence of the Rickettsiales bacterium “Montezuma”, a recently discovered bacterium, which has not been affiliated with any of the Rickettsiales families and forms a separate cluster in this order [44, 45]. Before this study, the Rickettsiales bacterium “Montezuma” had been found only in *I*. *persulcatus* ticks, whereas we for the first time detected it in *I*. *pavlovskyi* ticks.

The genus *Francisella* is composed of gram-negative intracellular parasites, in particular, the tularemia (*F*. *tularensis*) and septicemia (*F*. *philomiragia*) agents. The bacteria of this genus account for approximately half of the *D*. *reticulatus* microbiomes (both male and female) versus 0.1% of the microbiomes from the examined *Ixodes* tick species. Earlier investigations showed the presence of *Francisella*-like endosymbionts in *D*. *reticulatus* ticks [38] and a high infection rate of *D*. *variabilis* ticks with *F*. *tularensis*, though, this high rate of infection was inconsistent with the rare transmission of this disease via tick bites [46]. Meanwhile, the presence of *Francisella* spp. bacteria in the *I*. *persulcatus*, *I*. *ovatis* microbiomes was not demonstrated [30]. So, we could hypothesize that *Francisella* spp is a common member of microbiomes associated with different species of the *Dermacentor* genus.

The bacterial pathogens belonging to the genera *Borrelia*, *Ehrlichia*, and *Anaplasma* are known to be detectable in *Ixodes* ticks [1,2]. We also identified DNA sequences of the bacteria belonging to these genera in the pooled samples of *I*. *persulcatus* and *I*. *pavlovskyi* ticks with both *B*. *burgdorferi* s. l. and *B*. *miyamotoi* found in these two tick species. Recently, *B*. *miyamotoi* was isolated from *I*. *pavlovskyi* ticks inhabiting Hokkaido, Japan [47] and here we first report the presence of *B*. *miyamotoi* in *I*. *pavlovskyi* ticks in Siberia. These results indicated that *I*. *pavlovskyi* along with *I*. *persulcatus* could be a common tick vector for this pathogenic bacterium. No borrelias were detected in *D*. *reticulatus* microbiome, which is in agreement with some studies of these ticks in other regions [48]. Sometimes, borrelia DNA could be found in solitary *D*. *reticulatus* ticks, but this vector is not competent to transmit *Borrelia* spp. [14,49]. In our study, *D*. *reticulatus* ticks were collected in a sampling site where this tick species was dominated, while *I*. *persulcatus* and *I*. *pavlovskyi* ticks were rare species. Therefore, the possibility for *D*. *reticulatus* ticks to feed on the same reservoir host as ixodes ticks and acquire *Borrelia* spp. was low. DNA sequences of *A*. *phagocytophilum* were revealed in both *Ixodes* tick species as well. Notably, this is the first report of *A*. *phagocytophilum* and *Candidatus* Neoehrlichia mikurensis in *I*. *pavlovskyi* ticks. In *D*. *reticulatus* pooled samples, DNA sequences from bacteria belonging to the genus *Ehrlichia* were detected, which is the first discovery of these bacteria in this tick species. Further examinations are necessary to determine *Ehrlichia* genetic variants associated with *D*. *reticulatus* ticks.

In summary, 16S metagenomic profiling of the bacterial communities associated with *I*. *persulcatus*, *I*. *pavlovskyi*, and *D*. *reticulatus* ticks collected in the Novosibirsk region of Russia indicated that only several bacterial taxa with high abundance form the major share of microbiomes in all studied tick groups. This approach confirmed the results of long-term examination of bacterial pathogen occurrence in *I*. *persulcatus* ticks in Novosibirsk region and revealed that the spectrum of infectious agents found in the less-studied *I*. *pavlovskyi* and *D*. *reticulatus* ticks was wider than previously thought. Thus, this approach could be used for rapid assessment of the occurrence of medically important bacteria in tick populations inhabiting different natural biotopes and could therefore determine the epidemic danger of particular foci of infections transmitted by ticks.

## Supporting Information

S1 Fig MEGAN comparsion of taxonomic structure of tick groups. Samples in the tree were merged by tick species and gender. The height of the bars cohered to the log-scaled number of hits for each taxon.
(PNG)

S1 Table Share of all bacterial taxa in samples. Each sheet in the file describes share of taxa for specific taxonomic level: genus, family, order, class. Taxonimic level is given on the sheet name.
(ZIP)

## References

[1] Parola P, Raoult D (2001) Ticks and tickborne bacterial diseases in humans: an emerging infectious threat. Clin Infect Dis. 32: 897–928. 11247714 10.1086/319347

[2] Heyman P, Cochez C, Hofhuis A, van der Giessen J, Sprong H, Porter SR, et al (2010) A clear and present danger: tick-borne diseases in Europe. Expert Rev Anti Infect Ther. 8: 33–50. 10.1586/eri.09.118 20014900

[3] Dantas-Torres F, Chomel BB, Otranto D (2012) Ticks and tick-borne diseases: a One Health perspective. Trends Parasitol. 28: 437–446. 10.1016/j.pt.2012.07.003 22902521

[4] Bakhvalova VN, Rar VA, Tkachev SE, Matveev VA, Matveev LE, Karavanov AS, et al (2000) Tick-borne encephalitis virus strains of Western Siberia. Virus Res. 70:1–12. 11074120 10.1016/s0168-1702(00)00174-x

[5] Mikryukova TP, Moskvitina NS, Kononova YV, Korobitsyn IG, Kartashov MY, Tyuten Kov OY, et al (2014) Surveillance of tick-borne encephalitis virus in wild birds and ticks in Tomsk city and its suburbs (Western Siberia). Ticks Tick Borne Dis. 5: 145–151. 10.1016/j.ttbdis.2013.10.004 24380691

[6] Tkachev S, Panov V, Dobler G, Tikunova N (2014) First detection of Kemerovo virus in *Ixodes pavlovskyi* and *Ixodes persulcatus* ticks collected in Novosibirsk region, Russia. Ticks Tick Borne Dis. 5: 494–496. 10.1016/j.ttbdis.2014.03.003 24880473

[7] Beklemishev AB, Dobrotvorsky AK, Piterina AV, Ivanov ID, Nomokonova NY, Livanova NN (2003) Detection and typing of *Borrelia burgdorferi* sensu lato genospecies in *Ixodes persulcatus* ticks in West Siberia, Russia. FEMS Microbiol Lett. 227: 157–161. 14592703 10.1016/S0378-1097(03)00581-0

[8] Fomenko NV, Livanova NN, Chernousova NY (2008) Diversity of *Borrelia burgdorferi* sensu lato in natural foci of Novosibirsk region. Int. J Med. Microbiol. 298(S1): 139–148.

[9] Fomenko NV, Borgoiakov VIu, Panov VV (2011) Genetic features of *Borrelia miyamotoi* transmitted by *Ixodes persulcatus* . Mol Gen Mikrobiol Virusol. 2:12–17. 21786631

[10] Shpynov S, Fournier PE, Rudakov N, Raoult D (2003) "*Candidatus* Rickettsia tarasevichiae" in *Ixodes persulcatus* ticks collected in Russia. Ann N Y Acad Sci. 990: 162–172. 12860621 10.1111/j.1749-6632.2003.tb07358.x

[11] Shpynov S, Fournier PE, Rudakov N, Tarasevich I, Raoult D (2006) Detection of members of the genera *Rickettsia*, *Anaplasma*, and *Ehrlichia* in ticks collected in the Asiatic part of Russia. Ann N Y Acad Sci. 1078: 378–383. 17114745 10.1196/annals.1374.075

[12] Rar VA, Livanova NN, Panov VV, Doroschenko EK, Pukhovskaya NM, Vysochina NP, et al (2010) Genetic diversity of *Anaplasma* and *Ehrlichia* in the Asian part of Russia. Ticks Tick Borne Dis. 1: 57–65. 10.1016/j.ttbdis.2010.01.002 21771512

[13] Morozova OV, Cabello FC, Dobrotvorsky AK (2004) Semi-nested PCR detection of *Bartonella henselae* in *Ixodes persulcatus* ticks from Western Siberia, Russia. Vector Borne Zoonotic Dis. 4: 306–309. 15671737 10.1089/vbz.2004.4.306

[14] Rar VA, Fomenko NV, Dobrotvorsky AK, Livanova NN, Rudakova SA, Fedorov EG, et al (2005) Tickborne pathogen detection, Western Siberia, Russia. Emerg Infect Dis. 11: 1708–1715. 16318722 10.3201/eid1111.041195 PMC3367347

[15] Korenberg EI, Nefedova VV, Romanenko VN, Gorelova NB (2010) The tick *Ixodes pavlovskyi* as a host of spirochetes pathogenic for humans and its possible role in the epizootiology and epidemiology of borrelioses. Vector Borne Zoonotic Dis. 10: 453–458. 10.1089/vbz.2009.0033 19929222

[16] Chausov EV, Ternovoi VA, Protopopova EV, Kononova JV, Konovalova SN, Pershikova NL, et al (2010) Variability of the tick-borne encephalitis virus genome in the 5' noncoding region derived from ticks *Ixodes persulcatus* and *Ixodes pavlovskyi* in Western Siberia. Vector Borne Zoonotic Dis. 10: 365–375. 10.1089/vbz.2009.0064 19877811

[17] Romanenko VN (2005) The peculiarities of the biology of ticks inhabiting the environs of Tomsk City. Parazitologiia. 39: 365–370. 16316054

[18] Livanova NN, Livanov SG, Panov VV (2011). Characteristics of the distribution of ticks *Ixodes persulcatus* and *Ixodes pavlovskyi* at the border between the forest and forest-steppe zones in the territory near Ob River. Parazitologiia. 45: 94–103. 21874842

[19] Romanenko V, Leonovich S (2015) Long-term monitoring and population dynamics of ixodid ticks in Tomsk city (Western Siberia). Exp. Appl. Acarol. 10.1007/s10493-015-9879-2 25633264

[20] Karan LS, Ciccozzi M, Yakimenko VV, Lo Presti A, Cella E, Zehender G, et al (2014) The deduced evolution history of Omsk hemorrhagic fever virus. J Med Virol. 86: 1181–1187. 10.1002/jmv.23856 24259273

[21] Mediannikov O, Matsumoto K, Samoylenko I, Drancourt M, Roux V, Rydkina E, et al (2008) *Rickettsia raoultii* sp. nov., a spotted fever group rickettsia associated with *Dermacentor* ticks in Europe and Russia. Int J Syst Evol Microbiol. 58: 1635–1639. 10.1099/ijs.0.64952-0 18599708

[22] Rar V, Epikhina T, Suntsova O, Kozlova I, Lisak O, Pukhovskaya NM, et al (2014) Genetic variability of *Babesia* parasites in *Haemaphysalis* spp. and *Ixodes persulcatus* ticks in the Baikal region and Far East of Russia. Infect Genet Evol. 28: 270–275. 10.1016/j.meegid.2014.10.010 25460820

[23] Hudson ME (2008) Sequencing breakthroughs for genomic ecology and evolutionary biology. Mol Ecol Resour. 8: 3–17. 10.1111/j.1471-8286.2007.02019.x 21585713

[24] Chakravorty S, Helb D, Burday M, Connell N, Alland D (2007) A detailed analysis of 16S ribosomal RNA gene segments for the diagnosis of pathogenic bacteria. J Microbiol Methods. 69: 330–339. 17391789 10.1016/j.mimet.2007.02.005 PMC2562909

[25] Carpi G, Cagnacci F, Wittekindt NE, Zhao F, Qi J, Tomsho LP, et al (2011) Metagenomic profile of the bacterial communities associated with *Ixodes ricinus* ticks. PLoS One. 6:e25604 10.1371/journal.pone.0025604 22022422 PMC3192763

[26] Nakao R, Abe T, Nijhof AM, Yamamoto S, Jongejan F, Ikemura T, et al (2013) A novel approach, based on BLSOMs (Batch Learning Self-Organizing Maps), to the microbiome analysis of ticks. ISME J. 7: 1003–1015. 10.1038/ismej.2012.171 23303373 PMC3635243

[27] Vayssier-Taussat M, Moutailler S, Michelet L, Devillers E, Bonnet S, Cheval J, et al (2013) Next generation sequencing uncovers unexpected bacterial pathogens in ticks in western Europe. PLoS One. 8: e81439 10.1371/journal.pone.0081439 24312301 PMC3842327

[28] Menchaca AC, Visi DK, Strey OF, Teel PD, Kalinowski K, Allen MS, et al (2013) Preliminary assessment of microbiome changes following blood-feeding and survivorship in the *Amblyomma americanum* nymph-to-adult transition using semiconductor sequencing. PLoS One. 8: e67129 10.1371/journal.pone.0067129 23826210 PMC3691118

[29] Williams-Newkirk AJ, Rowe LA, Mixson-Hayden TR, Dasch GA (2014) Characterization of the bacterial communities of life stages of free living lone star ticks (*Amblyomma americanum*). PLoS One. 9: e102130 10.1371/journal.pone.0102130 25054227 PMC4108322

[30] Qiu Y, Nakao R, Ohnuma A, Kawamori F, Sugimoto C (2014) Microbial population analysis of the salivary glands of ticks; a possible strategy for the surveillance of bacterial pathogens. PLoS One. 9: e103961 10.1371/journal.pone.0103961 25089898 PMC4121176

[31] Masella AP, Bartram AK, Truszkowski JM, Brown DG, Neufeld JD (2012) PANDAseq: paired-end assembler for illumina sequences. BMC Bioinformatics. 13: 31 10.1186/1471-2105-13-31 22333067 PMC3471323

[32] Caporaso JG, Kuczynski J, Stombaugh J, Bittinger K, Bushman FD, Costello EK, et al (2010) QIIME allows analysis of high-throughput community sequencing data. Nat Methods. 7: 335–336. 10.1038/nmeth.f.303 20383131 PMC3156573

[33] Edgar RC (2010) Search and clustering orders of magnitude faster than BLAST. Bioinformatics. 26: 2460–2461. 10.1093/bioinformatics/btq461 20709691

[34] Wang Q, Garrity GM, Tiedje JM, Cole JR (2007) Naive Bayesian classifier for rapid assignment of rRNA sequences into the new bacterial taxonomy. Appl Environ Microbiol. 73: 5261–5267. 17586664 10.1128/AEM.00062-07 PMC1950982

[35] Oksanen J, Guillaume Blanchet F, Kindt R, Legendere P, Minchin PR, O’Hara RB, et al. (2013). vegan: Community Ecology Package. R package version 2.0–10.

[36] Dray S, Dufour A (2007) The ade4 package: Implementing the duality diagram for ecologists. J Stat Software 22: 1–20.

[37] Wang Y, Qian PY (2009) Conservative fragments in bacterial 16S rRNA genes and primer design for 16S ribosomal DNA amplicons in metagenomic studies. PLoS One 4: e7401 10.1371/journal.pone.0007401 19816594 PMC2754607

[38] Michelet L, Bonnet S, Madani N, Moutailler S (2013) Discriminating *Francisella tularensis* and Francisella-like endosymbionts in *Dermacentor reticulatus* ticks: evaluation of current molecular techniques. Vet Microbiol. 163: 399–403. 10.1016/j.vetmic.2013.01.014 23415475

[39] Cordaux R, Paces-Fessy M, Raimond M, Michel-Salzat A, Zimmer M, Bouchon D (2007) Molecular characterization and evolution of arthropod-pathogenic *Rickettsiella* bacteria. Appl Environ Microbiol. 73: 5045–5047. 17557851 10.1128/AEM.00378-07 PMC1951046

[40] Beninati T, Lo N, Sacchi L, Genchi C, Noda H, Bandi C (2004) A novel alpha-proteobacterium resides in the mitochondria of ovarian cells of the ticks *I*. *ricinus* . Appl Environ Microbiol. 70: 2596–2602. 15128508 10.1128/AEM.70.5.2596-2602.2004 PMC404433

[41] Rymaszewska A. (2007) Symbiotic bacteria in oocyte and ovarian cell mitochondria of the tick Ixodes ricinus: biology and phylogenetic position. Parasitol Res. 100: 917–920. 17226040 10.1007/s00436-006-0412-8

[42] Murrell A, Dobson SJ, Yang X, Lacey E, Barker SC (2003) A survey of bacterial diversity in ticks, lice and fleas from Australia. Parasitol Res. 89: 326–334. 12632173 10.1007/s00436-002-0722-4

[43] Alberdi MP, Nijhof AM, Jongejan F, Bell-Sakyi L (2012) Tick cell culture isolation and growth of *Rickettsia raoultii* from Dutch *Dermacentor reticulatus* ticks. Ticks Tick Borne Dis. 3(5–6): 349–354. 10.1016/j.ttbdis.2012.10.020 23140894 PMC3528949

[44] Mediannikov OIu, Ivanov LI, Nishikawa M, Saito R, Sidel'nikov IuN, Zdanovskaia NI, et al (2004) Microorganism "Montezuma" of the order Rickettsiales: the potential causative agent of tick-borne disease in the Far East of Russia. Zh Mikrobiol Epidemiol Immunobiol. 1: 7–13. 15024973

[45] Eremeeva ME, Oliveira A, Moriarity J, Robinson JB, Tokarevich NK, Antyukova LP, et al (2007) Detection and identification of bacterial agents in *Ixodes persulcatus* Schulze ticks from the north western region of Russia. Vector Borne Zoonotic Dis. 7: 426–436. 17767409 10.1089/vbz.2007.0112

[46] Goethert HK, Telford SR 3rd (2010) Quantum of infection of *Francisella tularensis tularensis* in host-seeking *Dermacentor variabilis* . Ticks Tick Borne Dis. 1: 66–68. 10.1016/j.ttbdis.2010.01.001 20563231 PMC2885736

[47] Takano A, Toymane K, Konnai S, Ohashi K, Nakao M, Ito T, et al (2014) Tick surveillance for relapsing fever spirochete *Borrelia miyamotoi* in Hokkaido, Japan. PLoS One 9: e104532 10.1371/journal.pone.0104532 25111141 PMC4128717

[48] Richter D, Kohn C, Matuschka FR (2013) Absence of *Borrelia* spp., Candidatus Neoehrlichia mikurensis, and *Anaplasma phagocytophilum* in questing *adult Dermacentor reticulatus* ticks. Parasitol Res. 112: 107–111. 10.1007/s00436-012-3110-8 22955502

[49] Lledó L, Gegúndez MI, Giménez-Pardo C, Álamo R, Fernández-Soto P, Nuncio MS, et al (2014) A seventeen-year epidemiological surveillance study of *Borrelia burgdorferi* infections in two provinces of northern Spain. Int J Environ Res Public Health. 11: 1661–1672. 10.3390/ijerph110201661 24487455 PMC3945560

