# Supplementary figures and images for: Comparative Metagenomic Profiling of Symbiotic Bacterial Communities Associated with Ixodes persulcatus, Ixodes pavlovskyi and Dermacentor reticulatus Ticks
Source: PLoS One. 2015 Jul 8;10(7):e0131413. doi: 10.1371/journal.pone.0131413 (PMC4496043; doi:10.1371/journal.pone.0131413)

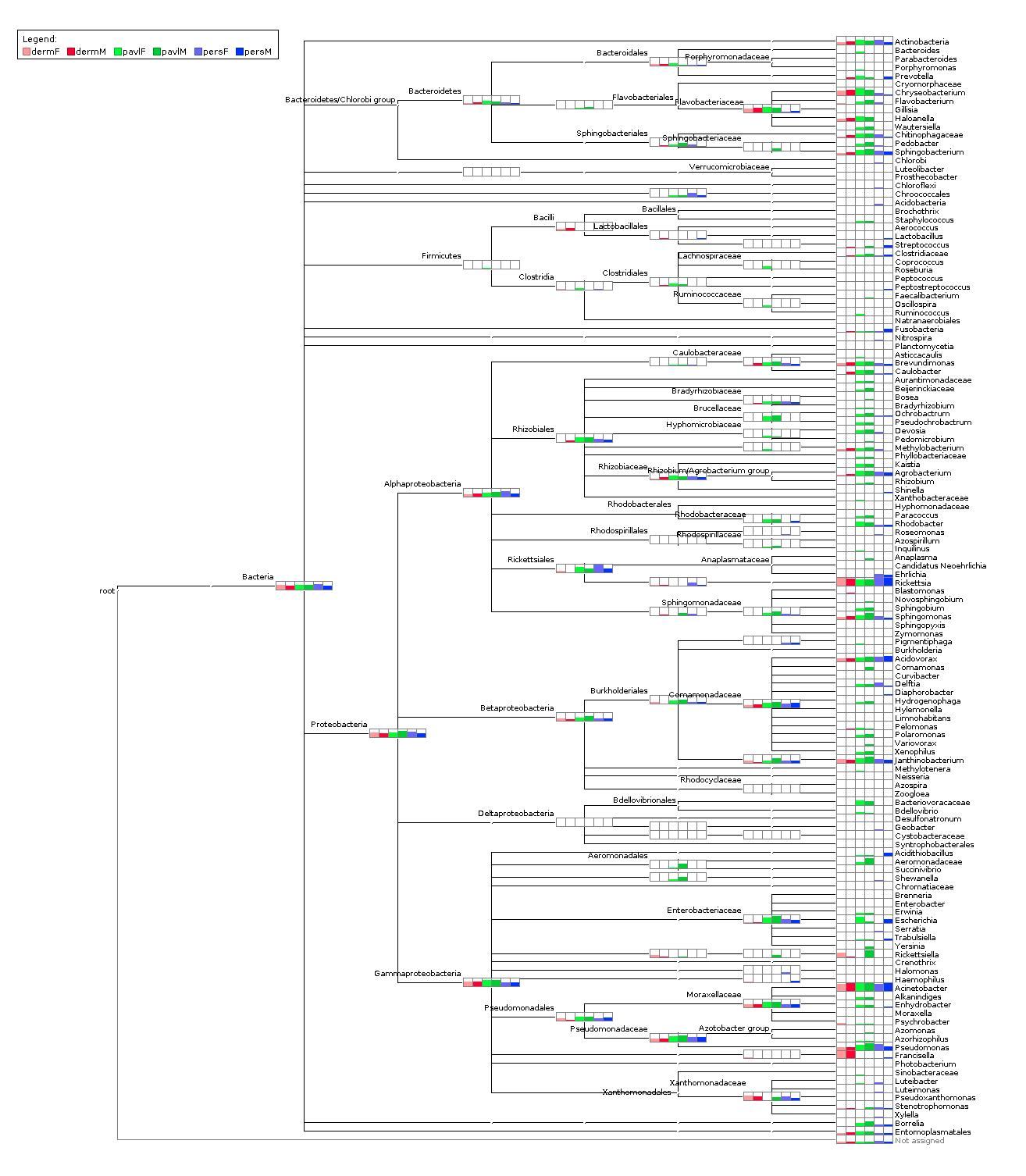

Supplement: S1 Fig — Samples in the tree were merged by tick species and gender. The height of the bars cohered to the log-scaled number of hits for each taxon. (PNG) [file pone.0131413.s001.png]
